# Current theoretical models fail to predict the topological complexity of the human genome

**DOI:** 10.3389/fmolb.2015.00048

**Published:** 2015-08-21

**Authors:** Javier Arsuaga, Reyka G. Jayasinghe, Robert G. Scharein, Mark R. Segal, Robert H. Stolz, Mariel Vazquez

**Affiliations:** ^1^Department of Mathematics, University of California, DavisDavis, CA, USA; ^2^Department of Molecular and Cellular Biology, University of California, DavisDavis, CA, USA; ^3^Division of Biology and Biomedical Sciences, Department of Medicine, Department of Genetics, The Genome Institute at Washington University in St. LouisSt. Louis, MO, USA; ^4^Hypnagogic SoftwareVancouver, BC, Canada; ^5^Department of Epidemiology and Biostatistics, University of California, San FranciscoSan Francisco, CA, USA; ^6^Department of Microbiology and Molecular Genetics, University of California, DavisDavis, CA, USA

**Keywords:** chromosome organization, DNA knotting, equilibrium globule, Hi-C, BFACF, lattice models

## Abstract

Understanding the folding of the human genome is a key challenge of modern structural biology. The emergence of chromatin conformation capture assays (e.g., Hi-C) has revolutionized chromosome biology and provided new insights into the three dimensional structure of the genome. The experimental data are highly complex and need to be analyzed with quantitative tools. It has been argued that the data obtained from Hi-C assays are consistent with a fractal organization of the genome. A key characteristic of the fractal globule is the lack of topological complexity (knotting or inter-linking). However, the absence of topological complexity contradicts results from polymer physics showing that the entanglement of long linear polymers in a confined volume increases rapidly with the length and with decreasing volume. *In vivo* and *in vitro* assays support this claim in some biological systems. We simulate knotted lattice polygons confined inside a sphere and demonstrate that their contact frequencies agree with the human Hi-C data. We conclude that the topological complexity of the human genome cannot be inferred from current Hi-C data.

## 1. Introduction

The 3-dimensional (3D) organization of the genome is a key functional component of the cell, and errors in this organization are associated with a wide range of diseases (Mitelman et al., 2007). Definitive, high-resolution visualizations of how genomes are packed have remained elusive, even for the simplest organisms, since genomes are highly condensed (e.g., Holmes and Cozzarelli, 2000). The simplest models for understanding the complexity of 3D chromosome configurations consist of random self-avoiding walks in confined volumes (also known as equilibrium globules). Equilibrium globules capture features such as overall knotting and linking complexity, of the 3D organization of the genome in dsDNA icosahedral bacteriophages and in trypanosomes but they fail to predict detailed topological properties (Simpson and Da Silva, 1971; Liu et al., 1981; Wolfson et al., 1985; Borst, 1991; Arsuaga et al., 2002, 2005; Blackstone et al., 2011; Diao et al., 2014). The accuracy of this model for higher organisms remains to be investigated. Eukaryotes are widely believed to possess topologically simple genomes, and to therefore deviate from the equilibrium globule. Pioneering work in microscopy led to the discovery of chromosome territories (reviewed in Cremer et al., 2014) and to the proposal of new models for genome organization (e.g., Kreth et al., 2004). These models rejected an overall random organization of the genome in favor of large [megabase (*Mb*) scale] or small [kilobase (*kb*) scale] loops (Yokota et al., 1995; Münkel et al., 1999). Chromosome Conformation Capture (CCC) methods such as 3C, 4C, and 5C facilitate the study of long-range interactions between genetic loci (Dekker et al., 2002; Dostie et al., 2006; Zhao et al., 2006). Other methods, such as Hi-C, TCC, and ChIP-3C, extend previous CCC techniques to allow for genome-wide identification of interactions (Fullwood and Ruan, 2009; Lieberman-Aiden et al., 2009; Kalhor et al., 2011). These assays provide an unprecedented opportunity for expanding our knowledge of 3D genomic organization in higher organisms and potentially for addressing questions of a topological nature. However, the data derived from such assays are complex and their preprocessing, analysis and interpretation remains challenging.

In the context of Hi-C analyses of human cell lines (described in Section 2.1), Lieberman-Aiden et al. (2009) proposed the fractal globule (Grosberg et al., 1988) as a possible model for the 3D organization of the human genome. The validity of this model for the 3D organization of the human genome remains a matter of debate (Bohn and Heermann, 2010; Rousseau et al., 2011; Barbieri et al., 2012). Fractal globules are self-avoiding polygons characterized by the following attributes: non-equilibrium, self-similarity, topologically trivial (i.e., unknotted) and globularity (i.e., every linear section of the genome is locally folded into globules). However, based on a variety of studies of DNA confinement one could argue that topologically complex conformations are highly likely (Liu et al., 1981; Ménissier et al., 1983; Wolfson et al., 1985; Arsuaga et al., 2002; Shimamura and Deguchi, 2002; Virnau et al., 2005; Micheletti et al., 2006; Blackstone et al., 2011; Diao et al., 2012, 2014). It is unclear if such topological complexity (e.g., knotting or interlinking of chromosomes) is in agreement with available Hi-C data. We here examine whether non-equilibrated knotted conformations can be consistent with the data presented in Lieberman-Aiden et al. (2009). To this end we implement an optimization algorithm that generates globular structures with fixed topology. This algorithm is based on the well established BFACF Monte-Carlo method, described in Section 2.2 (Madras and Slade, 1996). Our results show that knotted polygons some of which are connected sums with multiple copies of a single non-trivial knot, are consistent with the experimental data reported in Lieberman-Aiden et al. (2009). We therefore suggest that current analyses of Hi-C data are inconclusive as to the topological state of the genome.

## 2. Methods

### 2.1. Hi-C data and the fractal globule

In Hi-C experiments, genomic DNA is cross-linked and linearized into fragments using restriction enzymes. The ends of these crosslinked fragments are biotinilated and ligated. Biotinilated junctions, termed *contacts*, are purified and sequenced. The sequenced regions are mapped to their 1D genomic position and their contact frequency is determined. In this study we utilize the Hi-C data from human cell lines presented in Lieberman-Aiden et al. (2009). There, log-log plots of genomic distance vs. contact frequency revealed a linear relationship with a slope of −1.08 in the range from 500 *kb* to 7 *Mb*. The authors used Monte-Carlo computer simulations of fractal globules and of equilibrium globules to generate 3D reconstructions of the human genome. In the resulting log-log plots each point is an average of over 500 simulated conformations. The authors fitted a power law (in this case a straight line) for loci between 10 and 100 simulated monomers away. They found the slope corresponding to fractal globules (−0.993) to be in better agreement with that from experimental measurements (−1.08) than the slope for the equilibrium globule (−1.508). The authors also computed the end-to-end distance *R*(*s*) for a fragment of fixed contour length *s* averaged over 100 conformations and emphasized the differences between the two models. The slope of the average of these distances for the fractal globule was 0.27 while the slope for the equilibrium globule was 0.175.

### 2.2. Simulation data

#### 2.2.1. The BFACF algorithm

**Figure 1:**
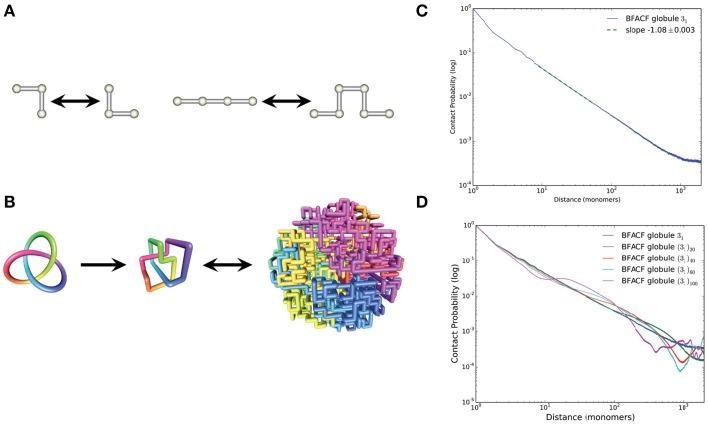
**Computational methods used to generate BFACF globules**. **(A)** BFACF moves: the 0-move (left) does not change the length of the conformation; the (+2)- and (-2)-moves (right) can add/remove an edge. **(B)** From left to right we illustrate a trefoil knot 3_1_ smoothly embedded in *R*^3^, a minimal step lattice realization of 3_1_, and the resulting BFACF globule. This BFACF globule is a 4000-step embedding of the knot within a sphere of radius 10.5 obtained using the modified BFACF algorithm described in Section 2. **(C)** Log-log plot of the contact probability as a function of contour length. The data are obtained as an average over 10,000 sampled BFACF globules with knot type 3_1_. The slope of the linear fit is in excellent agreement with the experimental data of Lieberman-Aiden et al. (2009). **(D)** Contact probability curves for connected sums of trefoils (_3_1_)*n*_ for *n* = 1, 20, 40, 60, 100, with slopes −1.085±0.003, −1.079±0.003, −0.919±0.011, −0.656±0.013, −0.558±0.035, respectively.

Our approach is based on, and extends, the BFACF algorithm. BFACF is a dynamic Monte Carlo method acting on the space of self-avoiding polygons in the simple cubic lattice (*Z*^3^) by performing one of the three local moves described in Figure 1A (Aragão de Carvalho and Caracciolo, 1983; Aragão de Carvalho et al., 1983; Madras and Slade, 1996). The acceptance probabilities for each move, denoted by *p*(0), *p*(2), and *p*(−2), are a function of the fugacity per bond *z*, where 0≤*z*≤*z*_0_. Within this range, the choice of *z* determines the average length of the generated lattice polygons. Going beyond this range causes the average polygon length to diverge. In BFACF the equilibrium conformations are sampled from a Boltzmann distribution (reviewed in Madras and Slade, 1996), and the ergodicity classes are the knot types (Janse van Rensburg and Whittington, 1991).

#### 2.2.2. Generation of BFACF globules

Inspired by the decondensation process that occurs at the end of metaphase and by the work of Rosa and Everaers (2008) and Lieberman-Aiden et al. (2009), we modified BFACF as an annealing algorithm to generate globules that filled a sphere of fixed radius *r* = 10.5 lattice units. We generated initial conformations by randomizing a polygon with minimal step number and the desired topology (Ishihara et al., 2012) using 10^7^ BFACF steps (with *z* = 0.1). We expanded the initial conformations to occupy 96% of the lattice vertices contained within a sphere of radius 11 (as in Lieberman-Aiden et al., 2009). Each annealing procedure was defined by a triplet [*p*(−2), *p*(0), *p*(2)] (Table 1). Given that the three parameters determine the rate at which the polygon grows within the confined region, we hypothesize that growth requires that favoring p(0) over p(-2) will take longer to fill the sphere. A total of 10,000 conformations were generated for each combination of parameters. We refer to these conformations as *BFACF globules*. For each generated conformation we estimated the contact probability between regions along the genome. A contact between two sites was scored as 1 for nearest neighbors in the lattice (i.e., points at distance 1) and as $1/2\sqrt{2}$ for diagonal adjacencies (points at distance of $\sqrt{2}$). There are different definitions and possibilities to compute the contact probability between two loci. We here define the contact probability between two sites at a fixed genomic distance by dividing the number of all the contacts at that distance by the total number of occupied vertices. The mean end-to-end distance for each conformation was obtained by computing the distance between pairs of points in the globule at a fixed genomic distance. To avoid any biases introduced by the initial conformation we used the BFACF algorithm (with *z* = 0.1) to generate different initial conformations for each run. The process of generating BFACF globules is illustrated in Figure 1.

**Table 1:** **Combination of parameters *p*(−2), *p*(0), *p*(+2) used to define the BFACF globules**.

	**p(-2) p(0) p(2)**	**Contact Prob. ±σ_*contact*_**	**Confidence interval**	***R*(*s*)±σ_*R*(*s*)_**
Fractal globule		−0.993		0.2763
Equilibrium globule		−1.508		0.1753
Experimental data		−1.08		
Unknot 0_1_	0.25 0.25 **0.25**	−1.0688±0.003	[−1.075, −1.063 ]	0.3559±0.002
	0.25 0.25 **0.30**	−1.0670±0.004	[−1.075, −1.059 ]	0.3555±0.002
	0.25 0.25 **0.40**	−1.0672±0.003	[−1.073, −1.061 ]	0.3553±0.002
	0.25 0.25 **0.50**	−1.0673±0.003	[−1.073, −1.061 ]	0.3553±0.002
	0.25 **0.10** 0.25	−1.0712±0.003	[−1.077, −1.065 ]	0.3558±0.002
	0.25 **0.20** 0.25	−1.0690±0.003	[−1.075, −1.063 ]	0.3554±0.002
	0.25 **0.30** 0.25	−1.0615±0.003	[−1.067, −1.055 ]	0.3546±0.002
	0.25 **0.40** 0.25	−0.9742±0.004	[−0.983, −0.966 ]	0.3439±0.003
	**0.10** 0.25 0.25	−1.0843±0.003	[−1.091, −1.078 ]	0.3565±0.002
	**0.20** 0.25 0.25	−1.0764±0.003	[−1.083, −1.070 ]	0.3558±0.002
	**0.30** 0.25 0.25	−1.0491±0.003	[−1.055, −1.043 ]	0.3531±0.002
	**0.40** 0.25 0.25	−1.1545±0.007	[−1.168, −1.141 ]	0.4105±0.003
Trefoil 3_1_	0.10 0.25 0.25	−1.0848±0.003	[−1.091, −1.078 ]	0.3584±0.001
5-torus knot 5_1_	0.10 0.25 0.25	−1.0862±0.003	[−1.093, −1.080 ]	0.3593±0.001
5-twist knot 5_2_	0.10 0.25 0.25	−1.0842±0.003	[−1.091, −1.078 ]	0.3596±0.001
9-torus knot 9_1_	0.10 0.25 0.25	−1.0860±0.003	[−1.093, −1.079 ]	0.3606±0.001
20 trefoils (_3_1_)20_	0.10 0.25 0.25	−1.0792±0.003	[−1.086, −1.073 ]	0.3514±0.002
40 trefoils (_3_1_)40_	0.10 0.25 0.25	−0.9190±0.011	[−0.941, −0.897 ]	0.3045±0.005
60 trefoils (_3_1_)60_	0.10 0.25 0.25	−0.6556±0.013	[−0.682, −0.629 ]	0.2590±0.005
100 trefoils (_3_1_)100_	0.10 0.25 0.25	−0.5584±0.035	[−0.628, −0.489 ]	0.1952±0.002

*We report on the slopes of the contact probability curves and the mean end-to-end distance curve. The standard error is included in columns 2 and 4. In addition we provide 95% confidence intervals for the slope of each contact probability curve. Parameter choices are shown in column 2. Changes in parameters from one row to the next are in bold*.

## 3. Results

Simulation results are shown in Table 1. The data are organized by columns in the following order: types of knots analyzed; combination of parameters [*p*(−2), *p*(0), *p*(2)] used for each knot type; slope of the contact probability curve in the range [9, 55] (± standard error σ_*contact*_); 95% confidence intervals for the contact probability data; and slope of the end-to-end distance *R*(*s*)±σ_*R*(*s*)_. The first three rows show the experimental and simulated data in Lieberman-Aiden et al. (2009).

To determine the combination of parameters [*p*(−2), *p*(0), *p*(2)] that could best reproduce the Hi-C data, we selected a randomly generated unknotted conformation. The values of [*p*(−2), *p*(0), *p*(2)] were systematically modified, starting with (0.25, 0.25, 0.25) as indicated in Table 1, rows 4–15. Note that [1−(*p*(−2)+*p*(0)+*p*(2))] is the probability of remaining in the current configuration. One can make a few qualitative observations on the effects of the annealing parameters [*p*(−2), *p*(0), *p*(2)]. First, the slope for *R*(*s*) remains almost constant, near the value estimated for the fractal globule (≈$\frac{1}{3}$) (Lieberman-Aiden et al., 2009), for a wide range of parameters. This suggests that it is the local folding, and not the knotted state of the conformation, that largely determines the mean end-to-end distance. Second, as *p*(2) increases, changes in the slopes are negligible for both the contact probability and *R*(*s*). Similarly, changes in slope when increasing *p*(0) were small for contact probability (and *p*(0) = 0.1, 0.2, 0.3) and for *R*(*s*). At *p*(0) = 0.40 the contact probability slope was closer to that observed for the fractal globule (−0.974±0.004). Last, we investigated the effect of *p*(−2). In this case, we observed an overall increase of the slope (decrease in absolute value) for *p*(−2) ∈ {0.1, 0.2, 0.3} with maximum variations of ≈0.036 and ≈0.0038 for contact probability and *R*(*s*), respectively. The combination (0.40, 0.25, 0.25) resulted in a sharp change of contact probability slope with values closely matching the equilibrium globule.

The parameters that better minimized the difference between the experimentally observed slope and the simulated slope for the unknotted conformation were (0.10, 0.25, 0.25). These parameters were then used to generate BFACF globules with knot types: torus knots (3_1_, 5_1_, and 9_1_), twist knot (5_2_), and connected sums of *n* trefoils, for *n* = 20, 40, 60, 100, which are denoted by (_3_1_)*n*_ = 3_1_#3_1_#…#3_1_. We refer the reader to Murasugi (1996) for an introduction to knot theory, including the definition of the connected sum, and to Brasher et al. (2013) for a table with relevant knot nomenclature. As Table 1 shows, the same combination of parameters gave very good approximations for the different knot types tested, including connected sums of up to 20 trefoils; beyond this value the slope of the contact probability decreased in absolute value, reaching values near −0.56.

Next we considered the knotted portion of each BFACF globule. Our preliminary data suggest that the knots are not localized. We analyzed two sets of 10^4^ conformations for knots 3_1_ and 9_1_ obtained with the optimal parameter combination (0.10, 0.25, 0.25). We cut each knot at a pair of points at distance 1 from each other and excised as much of the conformation as possible while still retaining the initial topology. The procedure was repeated multiple times on each conformation. The smallest knotted arc had 1012 edges for 3_1_ (i.e., 25.3% of the total length) and 1452 edges for the 9_1_ (i.e., 36.3% of the total length). Note that a minimal 3_1_ knot in *Z*^3^ has 24 edges (Diao, 1993) and thus a minimal 3_1_ within the BFACF globule would occupy 0.6% of the total length. In a connected sum, a minimal (_3_1_)*n*_ can be tied with 20*n* lattice steps thus occupying 0.5*n%* of the total length, e.g., a connected sum of 40 trefoils can occupy as few as 20% of the total length of the BFACF globule.

## 4. Discussion

The widespread and growing interest in the experimental characterization of 3D chromatin structure is driven by the underlying hypothesis that structure is tightly related to function. In particular, gene regulation and cancer-driving gene fusions are believed to be strongly influenced by the 3D organization of the genome (Mitelman et al., 2007). Generating high resolution reconstructions of genome architecture based on Hi-C data is the subject of much current research but involves many challenges including computational bottlenecks (e.g., Segal et al., 2014; Zhang et al., 2013).

Lieberman-Aiden et al. (2009) proposed that the fractal globule model, initially developed in Grosberg et al. (1988), provides an explanation for the folding of the human genome at the megabase scale. The authors chose the equilibrium globule as a competitive chromatin model. Based on comparing the fractal globule with the equilibrium globule, which produces mostly knotted conformations, the authors concluded that “fractal globules are an attractive structure for chromatin segments because they lack knots” (Lieberman-Aiden et al., 2009). Our study however shows that knotted conformations are consistent with the currently available Hi-C data. Furthermore, determining whether the knots in BFACF globules are localized or spread is important (e.g., Tubiana et al., 2011). Preliminary results on 3_1_ and 9_1_ suggest that the knotted portion of the BFACF globules is not localized. If this is a general trend then it implies that large-scale topological complexity is compatible with Hi-C data. We will explore this question, as well as the comparison to other models (Yokota et al., 1995; Münkel et al., 1999) in a future publication.

The topology of genome is a problem that we are just beginning to understand. Studies on lower organisms such as viruses and trypanosomes have revealed high levels of topological knotting and interlinking. In fact, theoretical studies of different polymer models have widely shown that the knotting and linking probability grows rapidly upon confinement (Arsuaga et al., 2002; Virnau et al., 2005; Micheletti et al., 2006; Blackstone et al., 2011; Tubiana et al., 2011). It is believed that organisms have evolved structural mechanisms that help reduce topological complexity, given that the action of topoisomerases alone would not be sufficient for this purpose (Sikorav and Jannink, 1994). However, the existence of knots or links in the genomes of higher organisms remains to be determined. The main objective of this work is not to argue for topological complexity in mammalian genomes. If there exist knots in the genome they are likely to be transient structures, however some of these knotted conformations could serve a biological purpose in gene regulation since topological complexity is associated with decreased biological function.

Motivated by the results and conclusions in Lieberman-Aiden et al. (2009), we have here performed an analysis of the effects of knotting on the slope of the contact probability of a polygon in the simple cubic lattice. We have used the transition probabilities of the BFACF algorithm to dynamically generate non-equilibrated conformations. Our results show that combinations of the annealing parameters [*p*(−2), *p*(0), *p*(2)] on a variety of knots can reproduce the contact data observed experimentally, indicating that current theoretical analyses of Hi-C data are insufficient to determine the topological structure of the genome.

### 4.1. Data sharing

Computer programs used in this work are available through the Knotplot software. Data generated in this study are available upon request from the authors of the paper.

## Author contributions

JA, MS, and MV designed research; JA, RJ, RGS, RHS, MS, and MV performed research; RGS, RHS, and RJ contributed new analytic tools; JA, RJ, RHS, MS, and MV analyzed data; and JA, MS, and MV wrote the paper.

## Funding

We thank the referees for their careful review of the manuscript. This research was primarily supported by NIH-R01GM109457 (JA, MV, and MS). Other support came from DMS1057284 (MV and RHS), DMS0920887 (RJ and RGS), CMB training grant GM 007067 (RJ). The authors thank B. McAuley, M. Puoukham, D. Koenig, and W. Wright for helpful discussions, M. S. Flanner for contributing scripting expertise in matlab, python, and c++, and Barbara Ustanko, ELS, for editorial assistance with this manuscript.

### Conflict of interest statement

The authors declare that the research was conducted in the absence of any commercial or financial relationships that could be construed as a potential conflict of interest.
